# The relationship between teacher professional identity and burnout amid the pandemic: A moderated mediation model

**DOI:** 10.3389/fpubh.2022.956243

**Published:** 2022-12-21

**Authors:** Yishan Lin, Moses A. Ameyaw, Qinhan Zhang, Binghai Sun, Weijian Li

**Affiliations:** ^1^College of Teacher Education, Zhejiang Normal University, Jinhua, China; ^2^College of Psychology, Zhejiang Normal University, Jinhua, China; ^3^Research Center of Tin Ka Ping Moral Education, Zhejiang Normal University, Jinhua, China; ^4^Key Laboratory of Intelligent Education Technology and Application of Zhejiang Province, Zhejiang Normal University, Jinhua, China

**Keywords:** perceived organizational support, professional identity, psychological resilience, teacher burnout, work engagement

## Abstract

**Background:**

Teacher burnout is affected by personal and social factors. COVID-19 has greatly impacted teachers' physical and mental health, which could aggravate teacher burnout.

**Purpose:**

Based on the JD-R model, this study aims to investigate the relationship between teacher professional identity (TPI) and job burnout during the COVID-19 pandemic, and examine the moderating roles of perceived organizational support (POS) and psychological resilience (PR) in these relationships among primary and secondary school teachers in China.

**Methods:**

A total of 3,147 primary and secondary school teachers participated in this study.

**Findings:**

Work engagement played a mediating role in the relationship between professional identity and burnout; when the POS and PR scores were high, the predictive coefficient of TPI on burnout was the largest.

**Originality:**

This study tested the mechanism underlying the relationship between TPI and burnout, and explored the protective factors of burnout.

**Implications:**

This study supports the applicability of the JD-R model during COVID-19 and provides ideas for teachers to reduce burnout.

## Introduction

Burnout refers to emotional exhaustion, a low sense of achievement, and a depersonalized state of psychological stress caused by an individual's inability to cope effectively with work pressure in their occupational field (1, 2). This phenomenon often occurs in many helping professions, such as doctors (3), policers (4), and teachers (5). Compared to university teachers, teacher burnout is more serious among primary and secondary school teachers in China (6) because they face increased pressure from students' academic achievements and teachers' titles. This phenomenon of teacher burnout among primary and secondary school teachers in China became more serious after the COVID-19 outbreak (7, 8). A study conducted by Sokal et al. indicated that teachers were increasingly exhausted during the pandemic (7). With the outbreak of COVID-19, schools switched from traditional offline teaching to online teaching, which resulted in many difficulties for teachers, such as a lack of classroom management, a decline in the quality of teaching content, and difficulty in tracking teaching results (9–11). Furthermore, teachers would face pressure from family (e.g., work-family conflict) because they need to stay home during most of the pandemic, which would aggravate the conflict between work and family, all of which would increase teachers' workload during the COVID-19 pandemic, finally leading to serious burnout (12). Teacher burnout not only affects students' motivation (13) and academic achievement (14) but also has a negative effect on teachers' mental health [e.g., depression, (15)]. Considering the serious burnout teachers face during the COVID-19 pandemic and the negative consequences of burnout, it is necessary to explore the protective factors of teacher burnout, its underlying mechanisms, and moderating factors.

## Literature review

### Teacher professional identity and burnout

Teacher professional identity (TPI) is defined as the beliefs, values, and commitments that an individual holds toward being a teacher (16, 17). Some studies have found that TPI is not only an important indicator for measuring the quality of teachers' work, but also helps to promote teachers' job satisfaction, motivation, and work commitment (18). Some studies found that burnout was negatively affected by professional identity (PI) among some service professions (19–22). For example, Chen et al. found that during the COVID-19 pandemic, the PI of college teachers was an essential factor affecting burnout (19). Social identity theory (SIT) can explain why PI affects burnout, which posits that individuals in society actively compare themselves with groups similar to their own (social comparison) to confirm whether they have received due recognition and respect in the group. Most studies that have been conducted on SIT in the workplace believe that occupational identity is a manifestation of social identity in the workplace, which means that occupational identity is part of social identity. Some scholars have proposed that PI refers to the attitudes, values, knowledge, beliefs, and skills that are shared with others within a professional group, and can affect how people interact, compare, and differentiate themselves from other professional groups in the workplace (23). From the perspective of social identity, PI is a collective concept constructed by individuals' sense of belonging, values, recognition, and acceptance of their groups, which emphasizes the decisive role of objective factors in PI (24). When an individual perceives that society's recognition of their career is not in line with their set expectations, the individual will often seek to change their occupational status (25, 26). Based on this theory, we might conclude that, if an individual does not recognize their career, they might generate turnover intention. Thus, this study posits that teachers' PI is negatively associated with teacher burnout.

### The mediating role of work engagement

Work engagement (WE) is a positive, well-rounded, work-related emotional state characterized by energy, dedication, and focus (27). Research has shown that PI is positively correlated with WE (28–30). Other researchers have found a significant correlation between a single dimension of PI and WE (31). For example, the research conducted by Wang et al. found that PI was positively correlated with work engagement among hotel employees (30).

Regarding the relationship between PI and burnout, some researchers believe that WE is an independent concept that is negatively associated with burnout. It involves the way a person devotes more time and energy to complete their tasks (32, 33). The Job Demand-Resource (JD-R) model emphasizes that WE and burnout are two different psychological states induced by job demands and resources at work. Job demands can lead to psychological energy consumption, which ultimately leads to negative results such as burnout. Job resources are predictors of the motivational processes that can promote WE. Other researchers, using empirical research, have found that WE and burnout were negatively correlated (34–36). For example, Hultell and Gustavsson found that job demand and resources were predictors of WE and burnout; job demand positively predicted burnout, while job resources positively predicted WE (35). Theoretical and empirical research has found that TPI may buffer teachers' burnout through WE. Therefore, this study suggests that WE mediates the relationship between TPI and burnout (H1).

### The moderating role of perceived organizational support

Perceived Organizational Support (POS) refers to whether individuals feel that the organization values their contributions, whether individuals are content with the attention they provide to the organization, and whether the organization, in this case, schools, cares about the basic interests of employees (37). Organizational support theory (OST) suggests that teachers' perceptions of organizations depend on how much the organization values them (37, 38). Teachers are more dedicated, loyal, and responsive when they feel that the organization genuinely cares about their welfare and needs (39). It can be seen that POS is a positive attitude toward the profession.

No previous study has examined the relationship between TPI, POS, and WE. However, Social Exchange Theory (SET) can provide a perspective. SET suggests that employees are willing to provide their best (e.g., positive attitudes and hard work) to their organization when they receive corresponding respect from the organization (40). Furthermore, studies have shown that POS is positively correlated with PI (41–43) and can improve teachers' work satisfaction (44, 45). POS reduces the negative effect of strain on WE (46). Therefore, POS may enhance the positive effects of PI on WE. Therefore, this study proposes that POS plays a moderating role in TPI and WE (H2).

### The moderating role of psychological resilience

Psychological resilience (PR) is an individual's ability to bounce back in the face of adversity, trauma, tragedy, or stress. It is a vital personal psychological resource in today's fast-paced, high-stress, and unpredictable work environment (47). Previous studies have shown that WE is positively correlated with PR (48–50). Lyu et al. found that during the COVID-19 pandemic, both the PR and organizational identity of medical staff positively impacted WE (49). Clark et al. argued that the higher the PR of medical staff, the higher their WE. According to the JD-R model, job engagement is negatively related to burnout; PR is a psychological resource that can alleviate negative psychological problems caused by work (51). Liu et al. found that during the COVID-19 pandemic, the PR of high school teachers had a significant negative predictive effect on burnout and turnover intention (52). Teachers with high PR will have better interpersonal relationships, a more satisfying sense of job competence and work efficiency, and can alleviate burnout. Therefore, this study posits that PR might play a moderating role in the relationship between WE and burnout (H3).

Previous studies have shown that teachers' PI, WE, POS, and PR have positive effects on preventing or reducing the level of teacher burnout (53–55). However, these studies generally examined the impact of a single factor on teacher burnout, ignoring the interaction effect of several variables on burnout. Variables interact with each other; that is, the function of the variables is conditional. Thus, it is necessary to explore the interaction effects of these variables. Furthermore, most previous research has discussed the influence of certain independent variables on teacher burnout (56, 57), but few have discussed their mediating mechanisms. Understanding the underlying mechanisms of teacher burnout is important for researchers to design interventions to prevent burnout. Therefore, it is necessary to explore the mediating mechanisms of teacher burnout.

### Theoretical basis

The JD-R model could be the theoretical basis of this study to explain teacher burnout. The JD-R model mainly assumes that job burnout is caused by an imbalance between job demands and resources and that many job resources can compensate for the impact of high job demands on job burnout (58). Job resources can be divided into four categories: material, conditional, personal, and energy-based resources (59). In particular, TPI and PR could be regarded as resources of personal character that could be used to help individuals resist stress, whereas POS could be regarded as conditional resources obtained from the external environment (59). WE and burnout are closely related and fundamentally different concepts (27). Research has shown that WE plays a mediating role in the relationship between job resources and burnout (60), and POS and PR play moderating roles in these relationships (46, 52). Based on the theoretical basis and empirical evidence, this study proposes a theoretical model.

Based on the JD-R model theory, we propose a theoretical model. We expect that our results will not only support this theory but also extend it. The JD-R model posits that resources could help individuals prevent or reduce their level of burnout. Resources can be divided into personal, organizational, and other resources. Although the JD-R model posits that resources are significant for preventing burnout, it does not consider the effects of different levels of resources (e.g., personal and organizational resources), especially when they interact with one another. In our study, we considered the effects of personal resources (i.e., TPI and PR) and organizational resources (i.e., POS), which might differ from those of previous studies.

### Research questions

In summary, this study aimed to explore the mechanisms underlying the relationship between TPI and burnout. Based on these theories and empirical evidence, we propose the following hypotheses:

*Hypothesis 1*: WE mediates the relationship between TPI and burnout.
*Hypothesis 2*: POS moderates the relationship between TPI and work engagement.
*Hypothesis 3*: PR moderates the relationship between WE and teacher burnout.

**Figure 1:**
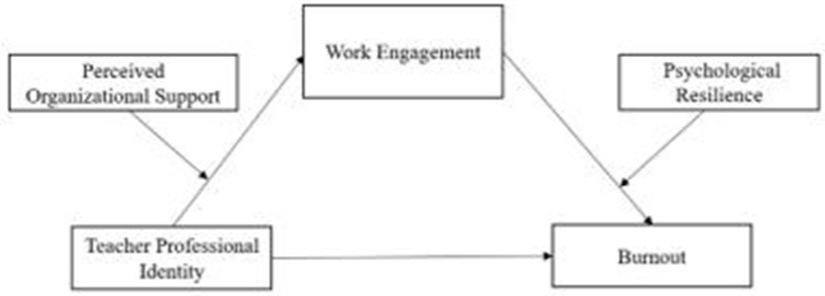
Hypothesized model of processes linking teacher professional identity and burnout, mediated by work engagement and moderated by perceived organizational support and psychological resilience.

The model is shown in Figure 1.

## Methods and materials

### Sample

This study was approved by the Ethics Committee of Zhejiang Normal University and performed in accordance with the Declaration of Helsinki and APA ethical standards. The survey was conducted over 3 months, from June to September 2021. Simple sampling was used, and 3,500 in-service teachers in a city in Zhejiang Province participated in this study. After excluding 353 questionnaires with incomplete responses, 3,147 valid questionnaires were included. There were 927 females and 2,220 males, with an average age of 39 years and a standard deviation of 8.74 years old. The average number of years of teaching was 17.90 years. Among the 3,147 valid questionnaires, 2,040 (64.82%) were pre-and primary school teachers, and 1,107 (35.18%) were secondary school teachers. A total of 2,856 (90.75%) teachers majored in teaching, and 291 (9.25%) majored in a non-teaching program.

### Measures

#### Burnout

Teacher burnout was measured using the Professional Quality of Life Scale designed by Stamm (61). This scale has three dimensions: burnout, compassion satisfaction, and secondary traumatic stress. The burnout subscale includes eight items. A five-point Likert scale (1 = never, 5 = very often) was used to ascertain participants' opinions. The higher the score, the stronger the burnout. In this study, Cronbach's alpha coefficient of the scale was 0.90.

#### Teacher professional identity

TPI was assessed using the Teachers' Professional Identity Scale by Wei et al. (62). The scale comprises four dimensions, namely, occupational values, role values, sense of occupational belonging, and professional behavior inclination, and eighteen items. All items are scored on a five-point Likert scale (1 = strongly disagree, 5 = strongly agree). In this study, Cronbach's alpha coefficient of the scale was 0.94.

#### Work engagement

WE was measured using the Utrecht Work Engagement Scale (UWES) by Schaufeli and Bakker (63), which consists of three dimensions: vigor, dedication, and absorption. The scale consists of nine items, and each item is scored on a five-point Likert scale (1 = never, 5 = always). In this study, Cronbach's alpha coefficient of the scale was 0.94.

#### Perceived organizational support

POS was measured using the Perceived Organizational Support Scale designed by Eisenberger et al. (64). The scale consists of nine items, and each item was scored according to a seven-point Likert scale (1 = strongly disagree, 7 = strongly agree). In this study, Cronbach's alpha coefficient of the scale was 0.91.

#### Psychological resilience

PR was assessed using the Psychological Capital Questionnaire designed by Zhang (65) for primary and secondary school teachers. The scale has nineteen items and four dimensions: resilience, self-confidence, hope, and optimism. A six-point Likert scale was used (1 = strongly disagree, 6 = strongly agree). In this study, Cronbach's alpha coefficient of the scale was 0.82.

## Procedures and data analysis

The data were collected electronically. First, a link to the questionnaire was sent to all participants *via* the Credamo platform. Second, the data were collated, and finally imported and analyzed using SPSS 21.0 (66). The data were analyzed as follows.

(1) Descriptive statistics and correlation analyses were performed on the main variables. (2) The SPSS PROCESS macro was used for mediation and moderation analysis with 5,000 bootstrapped samples. (3) Model 4 (PROCESS macro) was used to examine the mediating effect of job engagement between teachers' PI and job burnout. (4) Model 1 was used to examine the moderating effects of POS and resilience on teachers' PI and job engagement, as well as job engagement and burnout. (5) Model 21 was used to moderate the mediation analysis. A 95% CI was reported in our study. The CI in the Results section represents the 95% CI.

## Results

### Results of common method bias

Since the data collected by the questionnaire in this study were all from teachers' self-reports, there may be common methodological deviations. In this study, the Harman single-factor test was used as a statistical control. The results showed that the eigenvalues of 14 factors were >1, and the explanatory power of the first factor was 37.41%, which was less than the critical value of 40%, indicating that there were no serious problems resulting from common method bias in this study.

### Descriptive statistics and correlational analyses

The descriptive statistics are presented in Table 1. The results of correlational analyses showed that burnout was negatively correlated with TPI (*r* = −0.55, *p* < 0.01), WE (*r* = −0.67, *p* < 0.01), POS (*r* = −0.58, *p* < 0.01), and PR (*r* = −0.66, *p* < 0.01). TPI was positively related to WE (*r* = 0.63, *p* < 0.01), POS (*r* = 0.50, *p* < 0.01), and PR (*r* = 0.59, *p* < 0.01). Furthermore, the results found that WE was positively correlated with POS (*r* = 0.53, *p* < 0.01) and PR (*r* = 0.68, *p* < 0.01). These results indicate that TPI, WE, POS, and PR may be regarded as protective factors in reducing the level of burnout. The results are shown in Table 1.

**Table 1:** Means, standard deviations, and bivariate correlations among variables.

**Variables**	***M***	**SD**	**1**	**2**	**3**	**4**
TPI	4.50	0.49	–			
WE	3.90	0.78	0.63^**^	–		
BO	2.16	0.69	−0.55^**^	−0.67^**^	–	
POS	3.81	0.84	0.50^**^	0.53^**^	−0.58^**^	–
PR	4.90	0.79	0.59^**^	0.68^**^	−0.66^**^	0.51^**^

TPI, teacher professional identity; WE, work engagement; BO, burnout; POS, perceived organizational support; PR, psychological resilience.

^**^p < 0.01.

### Results of moderation effect of POS and PR

The results of the moderation effects of POS and PR after controlling for gender and age are shown in Table 2. Regarding the moderating role of POS, the results found that there was an interaction effect of TPI and POS on WE, indicating that POS played a moderating role in the relationship between TPI and WE [*B* = 0.06, *SE* = 0.03, CI = (0.01, 0.10), *p* < 0.05]. Further simple slope analysis showed that compared to the low POS (M-1SD), the predictive coefficient of TPI on WE increased from 0.74 [*SE* = 0.03, CI = (0.69, 0.79)] to 0.83 [*SE* = 0.03, CI = (0.75, 0.91)] when the POS score was high (M + 1 SD). As for the moderating role of PR, the results showed a significant interaction effect of WE and PR on burnout [*B* = −0.01, *SE* = 0.00, CI = (−0.01, 0.00), *p* < 0.05]. Further simple slope analysis showed that compared to the low PR (M – 1 SD), the predictive coefficient of WE on burnout increased from 0.34 [*SE* = 0.02, CI = (−0.37, −0.31)] to 0.41 [*SE* = 0.02, CI = (−0.44, −0.37)] when the PR score was high (M + 1 SD).

**Table 2:** Results of the moderation analyses.

**Y: WE**						**Y: BO**			
	***B***	**SE**	**95% CI**			***B***	**SE**	**95% CI**	
X: TPI	0.78^***^	0.03	0.73	0.83	X: WE	−0.37^***^	0.01	−0.40	−0.35
M: POS	0.29^***^	0.01	0.26	0.31	M: PR	−0.06^***^	0.00	−0.06	−0.05
X × M	0.06^*^	0.03	0.01	0.10	X × M	−0.01^**^	0.00	−0.01	0.00
Gender	0.15^***^	0.02	0.11	0.20	Gender	0.08^**^	0.02	0.04	0.12
Age	0.01^***^	0.00	0.01	0.01	Age	0.01^***^	0.00	0.00	0.01
Constant	3.42^***^	0.05	3.32	3.51	Constant	1.89^***^	0.04	1.81	1.97
	*R*^2^ = 0.49					*R*^2^ = 0.54			
	*F*_(5, 3, 141)_ = 602.58^***^					*F*_(5, 3, 141)_ = 739.64^***^			
	**Conditional effect of X on Y**					**Conditional effect of X on Y**			
	***B***	**SE**	**95% CI**			***B***	**SE**	**95% CI**	
M: M – 1 SD	0.74^***^	0.03	0.69	0.79	M: M – 1 SD	−0.34^***^	0.02	−0.37	−0.31
M: M + 1 SD	0.83^***^	0.04	0.75	0.91	M: M + 1 SD	−0.41^***^	0.02	−0.44	−0.37

CI, confidence interval; TPI, teacher professional identity; POS, perceived organizational support; WE, work engagement; PR, psychological resilience; BO, burnout.

*p < 0.05,

^**^p < 0.01,

^***^p < 0.001.

### Results of moderated mediation analysis

The results of the moderated mediation analysis, after controlling for gender and age, are shown in Table 3. The results showed that WE played a partial mediating role in the relationship between TPI and burnout [X-M: B = 0.78, CI = (0.73, 0.83), *p* < 0.001; M-Y: B = −0.33, CI = (−0.36, −0.30), *p* < 0.001; X-Y: B = −0.16, CI = (−0.20, −0.11), *p* < 0.001]. The results showed that POS played a moderating role in the relationship between TPI and WE, and PR played a moderating role in the relationship between WE and burnout. Further simple slope analysis showed that when the scores of POS and PR were high, the predictive coefficient of TPI on burnout through WE was −0.31; when the scores of POS and PR were low, the predictive coefficient of TPI on burnout was −0.22. This result indicated that when the POS and PR scores were high, the predictive effect of TPI on burnout through WE was the largest. The direct effect of TPI on burnout was −0.19. Thus, the indirect effect of WE accounted for 62.0% of the total effect.

**Table 3:** Results of the moderated mediation analysis.

	**M: WE**			**Y: BO**		
	***B***	**SE**	**95% CI**	***B***	**SE**	**95% CI**
X: TPI	0.78^***^;	0.03	0.73, 0.83	−0.16^***^;	0.02	−0.20, −0.11
W: POS	0.29^***^;	0.01	0.26, 0.31	–	–	–
X × W	0.06^*^	0.03	0.01, 0.10	–	–	–
M: WE	–	–	–	−0.33^***^;	0.02	−0.36, −0.30
V: PR	–	–	–	−0.05^***^;	0.00	−0.06, −0.05
M × V	–	–	–	−0.01^***^;	0.00	−0.01, 0.00
Gender	0.15^***^;	0.02	0.11, 0.20			
Age	0.01	0.00	0.01, 0.01			
Constant	−0.48^***^;	0.05	−0.57, −0.39	1.92^***^;	0.04	1.84, 2.00
	*R^2^* = 0.49			*R^2^* = 0.55		
	*F*_(5, 3, 141)_ = 602.58^***^;			*F*_(6, 3, 140)_ = 632.36^***^;		
				**The conditional indirect effect of X on Y**		
				***B***	**SE**	**95% CI**
		W: M – 1 SD	V: M – 1 SD	−0.22	0.02	−0.25, −0.18
			V: M + 1 SD	−0.28	0.02	−0.31, −0.24
		W: M + 1 SD	V: M – 1 SD	−0.24	0.02	−0.29, −0.20
			V: M+1SD	−0.31	0.03	−0.37, −0.25

CI, confidence interval; TPI, teacher professional identity; POS, perceived organizational support; WE, work engagement; PR, psychological resilience; BO, burnout.

^*^p < 0.05;

^***^p < 0.001.

## Discussion

Based on the JD-R model, this study explored the mediating role of WE in the relationship between TPI and burnout among Chinese primary and secondary school teachers and examined the moderating roles of POS and PR. This study reveals that when POS and PR are high, the predictive effect of TPI on burnout is greatest through WE. On the one hand, the organizational level should strengthen the affirmation of teachers' work so that teachers have a more positive attitude toward their work. On the other hand, it is necessary to provide full support to teachers to increase their positive psychological emotions, thereby reducing job burnout caused by long-term online teaching.

### The mediating role of WE between POS and burnout

According to the Pearson correlation results, the correlation between the three variables was significant at the 0.01 level. Specifically, the correlation coefficient between burnout and TPI was −0.55, that between burnout and WE was −0.67, and that between TPI and WE was 0.63. This result is consistent with previous research findings, that is, TPI had a negative effect on burnout (67), WE had a negative predictive effect on burnout (33), and TPI had a significant positive effect on WE (28).

This study demonstrated that WE played a partial mediating role (*p* < 0.001) in the relationship between TPI and burnout, supporting H1. This result is consistent with the findings of a study conducted by Zhang et al. on health inspectors. Their research found that PI not only directly affects burnout but also reduces the likelihood of burnout through WE (31). Therefore, teachers with strong professional identities are more engaged in their work, reducing the possibility of burnout.

Furthermore, previous studies have shown that individuals with high TPI have increased positive attitudes toward and greater commitment to their profession (68). Lack of TPI leads to teachers' stress and burnout (69). The positive state of WE can be used as a protective factor to alleviate burnout (34–36). Moreover, WE could be affected by TPI among primary and secondary school teachers. Teachers with a strong sense of PI are more engaged in their work, which reduces the possibility of burnout. WE could increase the mental state of teachers' high levels of energy, thereby reducing burnout. These statements support the hypothesis that WE mediates the relationship between TPI and burnout.

### The moderating roles of POS and PR

This study found that POS plays a moderating role in the relationship between TPI and WE, supporting H2. This result is similar to that of previous findings (70). This result can be explained as follows. Previous research has shown that WE can be positively affected by PI (16). When an individual has a high degree of recognition for their work, they will put more effort into it, leading to high WE. POS is defined as whether individuals feel that the organization values their contributions, whether individuals are content with the attention they provide to the organization, and whether the school cares about the basic interests of employees (37), which might play a moderating role in the relationship between teachers' PI and WE. There is an old saying in China that a gentleman will die for his confidant. If the organization respects employees and their values and cares about their lives, employees would be doubly engaged in their work. Previous studies have proven this (46, 71). For example, the research conducted by Zacher and Winter found that perceived organizational support was beneficial to employees' WE, and POS played a moderating role in the relationship between PI and WE (46). Thus, there is evidence to support that POS played a moderating role in the relationship between TPI and WE in our study.

This study found that PR played a moderating role in the relationship between WE and burnout among primary and secondary school teachers in China, supporting H3. Compared with low PR, the influence effect of WE on burnout increased from 0.33 to 0.40 for teachers with high PR. The results of this study are consistent with those of previous studies (52, 72, 73). For example, the research conducted by Liu et al. found that teachers with high mental toughness can better adjust and overcome difficulties when facing the problem of burnout caused by the pandemic and reduce the problems caused by burnout to a certain extent (52). In teachers' daily lives, they would face many difficulties from family, students' parents, and school managers, which would aggravate the level of burnout (74). PR is an internal positive psychological resource that can help individuals successfully cope with difficulties and adapt to stress. Therefore, teachers with increased WE can reduce burnout, and teachers with good PR will suffer from less burnout.

Rather than testing the JD-R model, our research supported the JD-R model to a certain degree. First, the complete JD-R model posits that burnout was a result of an imbalance between job resources and demands. However, our research only explored the effect of job resources on reducing the level of burnout and did not explore the effect of job demands on burnout; thus, it is difficult to say that our research tested the JD-R model. We have added this to the limitations of this study and future directions. Future research could simultaneously test the effects of job demands and resources on teacher burnout. Second, the theoretical hypothesized model in our research is posited based on the JD-R model. Analysis of the data revealed that the hypothesized model was feasible, supporting the JD-R model to some degree (i.e., these variables could be regarded as job resources that affect job burnout). Based on these two reasons, we believe that we did not test the JD-R model, but support it to a certain extent through relevant data analysis.

In this study, teachers' PI, POS, and PR were all work resources that played a protective role in the occurrence of teacher burnout. Regarding the mediating role of WE, previous studies have shown that burnout is closely related to WE, and WE is positively correlated with teachers' PI. Moreover, previous research has found that burnout is negatively related to other work resources, such as self-efficacy (75), empathy (76), organizational culture (77), and emotional intelligence (78).

### Implications

From the perspective of social organization and personal psychological resources, the following suggestions are made. First, to increase perceived social support, there is a need to organize training sessions for school administrators to strengthen their understanding of online teaching principles and quality monitoring. Teachers are also encouraged to engage in online teaching. Online teaching during the pandemic is not only a disadvantage but also increases the ability of teachers to teach online. Therefore, teachers should engage in online teaching with a positive attitude. Moreover, to alleviate burnout, support from external work resources such as POS can be strengthened to ensure the normality of online teaching. In summary, schools should strive to improve the service capabilities of their teaching environment, such as ensuring that teachers reorganize teaching equipment during the teaching process and that the teaching environment is comfortable. This will result in a better teaching platform and provide necessary support tools for teaching and learning. At the same time, schools need to build a teacher professional development community to support teachers in reducing professional burnout under the conditions of long-term online teaching.

### Limitations and future directions

This study has some limitations. First, the data we collected was purely correlational, collected at a single time point, and with no experimental manipulation or random assignment, which resulted in difficulties in inferring the causal relationship. Meanwhile, one drawback of cross-sectional data that was used to test mediation effects was that it was a lack of control for prior levels of variables. Thus, the results of mediation and moderation analysis should be interpreted with caution, which refers to the causalities implicitly implied by the arrows in the mediation model and that all evidence for causation comes from either the theoretical reasoning or the existing empirical findings. Future research could adopt an experimental design or longitudinal design to test the causal relationship between these variables. Second, the large sample size of this study did not apply nationwide. The study was conducted only in Zhejiang province. Future researchers could consider other variations, such as teachers at different levels (preschool and high school teachers). Furthermore, this study can be replicated in other provinces in China. Third, our research only explored the effect of job resources on burnout, ignoring the effect of job demands on burnout, which resulted in difficulties in testing the JD-R model. Future research could simultaneously consider the influence of job demands and resources on burnout.

## Conclusion

Based on the JD-R model, this study examined several protective factors that can help reduce burnout among teachers at the organizational and individual levels. These findings suggest that WE mediates the relationship between TPI and burnout. POS moderated the relationship between TPI and WE, while PR moderated the relationship between WE and burnout. Moreover, this study provides suggestions that can help overcome the problem of burnout among primary and secondary school teachers in China during the COVID-19 pandemic. It is worth noting that, although most schools have already started online teaching, it is unclear whether online teaching will still be part of daily teaching after the pandemic. However, online teaching is an important direction for future college education reform, and this study aims to provide an organizational reference for the development of online teaching in the future.

## Data availability statement

The raw data supporting the conclusions of this article will be made available by the authors, without undue reservation.

## Ethics statement

The studies involving human participants were reviewed and approved by the Ethics Committee of Zhejiang Normal University. The patients/participants provided their written informed consent to participate in this study.

## Author contributions

YL and BS: conceptualization. YL and QZ: methodology. BS and WL: validation. YL and QZ: resources. YL: writing—original draft preparation and funding acquisition. YL and MA: writing—review and editing. QZ and WL: supervision. BS: project administration. All authors contributed to the article and approved the submitted version.
